# “We Do Not Seem to Have Geriatric Wards”: A Qualitative Analysis of Gaps in Healthcare Access Among Older Patients in Addis Ababa, Ethiopia

**DOI:** 10.1002/hsr2.71449

**Published:** 2025-11-05

**Authors:** Kirubel Manyazewal Mussie, Bettina Zimmermann, Bernice Simone Elger, Jenny Setchell, Mirgissa Kaba

**Affiliations:** ^1^ Institute for Biomedical Ethics University of Basel Basel Switzerland; ^2^ Addis Center for Ethics and Priority Setting Addis Ababa University Addis Ababa Ethiopia; ^3^ Institute of History and Ethics in Medicine, School of Medicine School of Social Sciences, Technical University of Munich Munich Germany; ^4^ Center for Legal Medicine University of Geneva Geneva Switzerland; ^5^ School of Health and Rehabilitation Sciences The University of Queensland Brisbane Queensland Australia; ^6^ School of Public Health, Addis Ababa University Addis Ababa Ethiopia

**Keywords:** ageing, ethical issues, geriatric care, health professionals, older adults

## Abstract

**Background and Aims:**

Access to quality healthcare for older patients is a challenge worldwide, particularly in several African countries. As traditional support systems for older adults are weakening across the continent, older adults are increasingly relying on formal health systems that are not prepared enough to meet their needs. The aim of this study was to investigate the challenges of accessing healthcare among older patients in Addis Ababa, Ethiopia, from the perspective of older patients and healthcare professionals.

**Methods:**

Semi‐structured qualitative interviews were conducted with 20 older adults (60 years and older) and 26 health professionals, including physicians and nurses, purposively selected from health facilities in Addis Ababa, Ethiopia's capital. The interviews were audio recorded, transcribed verbatim and analysed using the reflexive thematic analysis approach.

**Results:**

Three themes were produced from our analysis of the data. First, participants stated that the physical and material infrastructure of health facilities was not convenient for providing specialized care for older patients. Second, they reported that specialized care services for older patients were highly limited or even absent. Third, they underlined limited geriatric expertise among health professionals as an additional challenge.

**Conclusion:**

The lack of specialized care for older patients in Ethiopia leaves this population vulnerable to increased health challenges and raises ethical concerns about justice in healthcare distribution. The findings can inform preparedness and health policy efforts aimed at improving the well‐being of older adults in Ethiopia and similar contexts. Additionally, there is a need to enhance geriatric training among healthcare professionals and establish departments specifically designed for geriatric care in health facilities.

## 1 Introduction

In 2015, 900 million people were over the age of 60 worldwide and this number is expected to reach two billion by 2050 [1]. 80% of this increase is projected to come from low‐ and middle‐income countries [1]. In Ethiopia, the older population accounted for 5.2 million in 2015 and is expected to reach 8.4 million by 2030 [2]. Globally, the older population is suffering from disability and death due to, for example, chronic health conditions and infectious diseases [3, 4, 5, 6]. Disease burden among older adults is also a serious public health concern in several sub‐Saharan African countries [7]. This challenge was exacerbated by the novel coronavirus disease 2019 (COVID‐19), which disproportionately affected the older population around the globe [8, 9, 10, 11]. Growing healthcare gaps imply a lack of universal access to healthcare which, according to the World Health Organization (WHO), requires that “all people have access to the full range of quality health services they need, when and where they need them, without financial hardship” and “covers the full continuum of essential health services, from health promotion to prevention, treatment, rehabilitation and palliative care” [12].

Although meeting the growing health needs of older adults is a public health challenge in high‐income countries as well [13], this gap is more concerning in the region of Africa, where health systems have limited readiness for geriatric care [14, 15]. However, there have been efforts in the region to address the health needs of older adults. For example, one major regional achievement is the African Union (AU) Protocol on the Rights of Older People ratified by the AU in 2016, which underlines that state parties should ensure healthcare access to older adults [16]. There have also been some notable policy efforts in Ethiopia to meet the needs of older adults. For example, Ethiopia is one of the three African countries (after Lesotho and Benin) to ratify the AU Protocol on the Rights of Older People in July 2020, which, according to HelpAge International, is a decision that will “help the country make deliberate and systematic efforts towards protecting the dignity and rights of older people” [17]. Other notable efforts include the issuance of the Developmental Social Welfare Policy in 1996 (replaced by the Social Protection Policy in 2014) [18] and the 10‐year National Plan of Action on Older Persons developed in June 2006 [19]. Despite these and other efforts not described in detail here, older adults in Ethiopia continue to face health and socioeconomic challenges [20, 21, 22, 23]. Geriatric wards are absent at the hospital level and most of the health sciences curricula in Ethiopia do not include gerontology training due to, among others, limited awareness about the field [24].

Healthcare delivery in Ethiopia is mainly structured into three tier‐system—primary, secondary, and tertiary—and is characterized by a combination of public and private providers. The current primary healthcare system in Ethiopia has been in place for the last three decades and is considered a model in Africa [25]. However, there are still challenges in ensuring healthcare coverage across the country. For example, there are significant variations in healthcare access and quality of services between urban and rural areas, with urban areas being much better off [26, 27]. The rate (per year) of utilization of healthcare services in urban areas is higher than the rural regions by 91% [28]. However, urban areas (such as Addis Ababa, the country's capital) are still way behind in getting adequate healthcare coverage [29].

In terms of healthcare financing, Ethiopia primarily uses an out‐of‐pocket payment system, although community‐based and social health insurance schemes have been introduced in recent years [30]. However, coverage remains limited, especially for older adults who are often retired or economically dependent. Most patients, including older ones, are required to pay directly for outpatient and inpatient services, diagnostics, and medications at public and private facilities. Reimbursement mechanisms are generally weak, particularly for patients without insurance [31]. This broader financing structure contributes to the economic dependency and burden frequently described in older patients' healthcare experiences.

Despite the fast increase in the older population, the deliberation regarding universal access to specialized care among older patients in Ethiopia, in particular, and Eastern Africa [32] in general is limited. Research about the care of older adults in Ethiopia has so far focused on basic services in nursing homes [33, 34], quantitative studies assessing determinants of healthcare‐seeking behavior among rural older adults [35, 36], or the life challenges of older people in the community in general [22, 37]. None of these studies addresses the gaps in healthcare facilities to provide specialized care for older patients and the experiences of older patients facing these challenges. Thus, this study aims to contribute towards addressing that gap by exploring the structural, professional, and experiential challenges older patients face in accessing healthcare in Ethiopia. In particular, the study seeks to examine how aspects such as geriatric training, health facility infrastructure, and the inclusion of older adults in health priorities influence access to care for older adults in Addis Ababa.

## 2 Methods

### 2.1 Study Design

We employed an exploratory qualitative design to investigate the challenges of healthcare access for older adults in Ethiopia, a topic with limited prior research in this setting. This approach was chosen to allow for an in‐depth understanding of a complex and context‐specific issue, especially given the multifaceted barriers older patients may face within the healthcare system. Qualitative inquiry, particularly when exploratory in nature, is well‐suited for uncovering the perspectives, meanings, and lived experiences of individuals in ways that structured methods might miss [38]. By prioritizing open‐ended inquiry, we sought to illuminate not just individual challenges but also the broader social and institutional dynamics that shape access to geriatric care in Ethiopia. This design enabled us to generate grounded insights that can inform future policy, practice, and research in similar low‐resource contexts.

### 2.2 Participants

We interviewed 20 older adults and 26 health professionals recruited using purposive and snowball sampling techniques [39]. The lead author (KMM) recruited all participants in person and with the assistance of medical directors at the health facilities. For the older adult participants, the inclusion criteria were: age (60 years and older), the ability to communicate and consent [40], and having a medical care experience (past and/or present) in Ethiopia. We defined old age as 60 years and above, following the UN definition of older adults in sub‐Saharan African countries [2] and the retirement age of 60 in Ethiopia [19]. The health professional participants were employees in healthcare facilities during the interview period and had experience providing healthcare for older adults in healthcare facilities in Ethiopia. Our health professional participants were recruited from two private and four governmental health facilities (from both outpatient and inpatient departments) in Addis Ababa, Ethiopia's capital, which were selected to increase healthcare facility diversity and hence to include a higher range of perspectives [41].

### 2.3 Backgrounds of the Researchers

The research team brings a multidisciplinary background that enriched the design, interpretation, and reflexivity of this study. KMM holds a PhD in biomedical ethics and has an interdisciplinary background in biomedical ethics, international community health, and social work. At the time of the study, he was a doctoral researcher at the Institute for Biomedical Ethics Basel (IBMB), with experience in qualitative research, ethics consultation, and health policy analysis. He was supervised by BSE, MK, and JS. BZ is a postdoctoral fellow at the Institute for Biomedical Ethics in Basel and the Technical University of Munich, with research expertise in bioethics and science communication. BSE is a professor of bioethics and head of the IBMB, with a dual background in medicine and theology and extensive experience in teaching, clinical ethics, and health law. JS is a senior research fellow in physiotherapy at The University of Queensland and Director of SocioHealthLab. She has a background in psychology and physiotherapy, and expertise in sociocultural approaches to healthcare. MK, a professor of public health at Addis Ababa University, has a deep contextual understanding of the Ethiopian healthcare system based on decades of research in public health, cultural epidemiology, and community engagement. The diversity in expertise and geographic perspective among the team strengthened the study's analytical depth and credibility.

### 2.4 Data Collection

Semi‐structured interviews were conducted between 3rd March and 28th November 2021 by KMM following an open‐ended and conversational approach [42]. The interview language was Amharic, which is Ethiopia's official language and the interviewer's first language. The first author prepared the interview guides based on discussions with project supervisors and conducted two pilot interviews (one with each participant group) to check that the interview guides were clear and open‐ended enough for participants [43]. The pilots were not included in the analysis. Following the pilots, we made minor changes such as elaborating on some questions and revising the Amharic translations of some ethical concepts.

The interview duration was approximately an hour on average (ranging from 15 to 107 min), and the questions focused on investigating how convenient the physical setup of health facilities is to provide geriatric care and the availability of specialized care services for older patients (see Additional file 1). Participants were also asked to describe the most common or current health conditions they experienced (for older patients) or observed in their clinical practice (for health professionals), to understand the nature of demand for geriatric care. Data collection was concluded when data saturation was reached, defined here not as a universal stopping point but as the moment when no substantially new patterns or concepts emerged from ongoing analysis, and when sufficient thematic richness had been achieved to adequately address the exploratory research aim [44]. All interviews were audio recorded except for two participants (one from each participant group) who did not consent to voice recording. The interviewer took notes on the interviews with these participants. KMM transcribed the audio recordings verbatim into English and anonymized them. MK and KMM, both native Amharic speakers, checked the transcripts for quality and translation accuracy.

### 2.5 Data Analysis

We used Braun and Clarke's six‐step reflexive thematic analysis approach, where coding is open and themes are created during the process of the analysis (inductive approach) [45], applying their understanding of thematic analysis as a collection of sometimes conflicting approaches to understanding data [46]. We entered the transcripts into the MAXQDA‐2022 software for storage and analysis of the transcripts [47]. The analysis was done separately for each participant group (older adults receiving care and health professionals providing care) since the questions were different and the participant groups are quite different from each other to make a comparative analysis. However, we indicated in the results when there were similarities or differences in similar questions or ideas.

KMM, the first author, began by openly coding a subset of transcripts from each group (6 in total) to develop a preliminary coding frame. This preliminary frame served as a flexible and evolving guide rather than a fixed structure. As further transcripts were analysed, new codes were added and existing ones revised to ensure the coding process remained sensitive to the data and aligned with the inductive orientation of the study. While KMM conducted the coding independently, feedback was regularly sought from MK, BSE, and JS. These co‐authors reviewed the preliminary coding frame and later iterations of the themes, and they provided critical input on clarity, code definitions, and conceptual framing. Their comments helped refine the thematic structure and ensured interpretive rigor. Final themes were developed by KMM based on all coded data and shared with BZ for further reflection and refinement.

### 2.6 Ethical Considerations

This study received ethical approval from the Ethics Committee of the University of Basel (Switzerland) and the research ethics committee at the City Government of Addis Ababa Health Bureau (Ethiopia). All interviewees received a study information leaflet from KMM. KMM and participants verbally discussed the contents of the information sheet, and participants were allowed to ask questions. Then, participants signed and returned the consent forms, and KMM conducted all the interviews in person at the health facilities. Study participants did not receive monetary compensation as they did not face any financial costs due to their participation [48, 49].

## 3 Results

**Figure 1:**
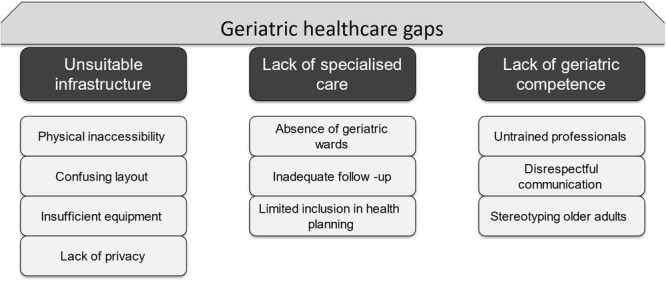
Thematic map of geriatric healthcare gaps.

We identified three major themes through reflexive thematic analysis that reflect the major gaps in Ethiopian healthcare regarding specialized care for older patients: (1) unsuitable infrastructure, (2) lack of specialized care, and (3) the lack of geriatric expertise among healthcare professionals. Each of these themes includes subthemes illustrating the reasons for these gaps and the consequences as perceived by participants (see Figure 1).

Participants' demographic characteristics are presented in Table 1. The older adults were between the ages of 60 and 87 years, with most being in the younger age range. There were substantial differences among older adult participants in terms of various demographic characteristics, such as socioeconomic status and health conditions. For example, the majority (65%) of the older adult participants were economically dependent on their families or communities (including for their health expenses). In contrast, the remaining 35% had sufficient financial means of their own to cover their health and living expenses in general. In the case of health professional participants, there were variations in terms of their professional background. They were predominantly nurses (46.1%), followed by general practitioners (19.2%), internists (11.5%), public health officers (7.7%), and other professionals. Moreover, they came from different healthcare facilities, such as government tertiary hospitals (6, 23%), government primary healthcare centers (9, 34.6%), and private hospitals (11, 42.3%).

**Table 1:** Demographic information of participants.

Older adults			
Participant ID	Age	Underlying health conditions*	Occupation
OP1	65	Chronic leg injuries, stomach ulcer, diabetes	No
OP2	86	Joint pain, hypertension, urinary tract infection	No
OP3	63	Diabetes, glaucoma	Self‐employed
OP4	87	Joint pain, lung problem (continuous coughing)	No
OP5	75	Thyroid disease, hypertension, vision problems, diabetes	No
OP6	68	Diabetes, hypertension, joint pain	No
OP7	63	Hypertension, hypertension, cardiovascular diseases	No
OP8	84	Joint pain, gastritis, chronic kidney disease, eye problems	No
OP9	74	Joint pain, stomach ulcer	No
OP10	64	Stomach ulcer	Physician
OP11	76	Leg injury, hypertension, joint pain	No
OP12	65	Back pain, diabetes	Self‐employed
OP13	62	Diabetes, hypertension, joint pain	Self‐employed
OP14	64	Migraine	Self‐employed
OP15	61	Joint pain	Pastor
OP16	60	Diabetes, stomach ulcer	Self‐employed
OP17	75	Joint pain, chronic kidney disease	No
OP18	69	Liver disease, gallbladder stone, hypertension	No
OP19	60	Back pain, migraine	Factory worker
OP20	77	Joint pain, thyroid disease	No
Gender: 11 F, 9 M Total: 20			

Health professionals			
Profession			Number of participants and percentage
Nurse			12 (46.1%)
General Practitioner			5 (19.2%)
Internist			3 (11.5%)
Public health officer			2 (7.7%)
Psychiatrist			1 (3.8%)
Neurologist			1 (3.8%)
Cardiologist			1 (3.8%)
Health officer			1 (3.8%)
Gender: 16 F, 10 M Total: 26			

*Note:* This table is adapted from a previously published article from the same research project [50]).

* The underlying health conditions were as reported by participants in the interviews and not taken from hospital records.

### 3.1 Unsuitable Infrastructure for Older Patients

All participants voiced their concerns regarding the inadequacy of health facilities to provide the needed quality of healthcare for older patients (Table 2). The majority of both older adults and health professionals broadly discussed the challenging infrastructure of existing healthcare facilities for older patients. For example, some healthcare facility buildings had only stairs but no lifts, which was particularly mentioned by participants recruited from government health facilities rather than the private. This was reported as a challenge for older patients, particularly for those with more mobility challenges. Health professionals also noted that the absence of lifts is challenging for older patients.

**Table 2:** Illustrative quotes for Theme 1: Unsuitable infrastructure for older patients.

Participants	Quotes
OP12	When I walk up the stairs, something pinches my leg, and that hurts me until I feel the pain up to my lower back area.
HP24	Not having a lift is a big problem for older patients.
HP22	There was this older man who came alone and fell and was hurt. […] The pathways in this facility are designed for a normal person, not an elderly individual. It has slippery stairs, and such people might fall.
OP1	I have suffered because of that. I have gone up and down and here and there in the compound. And there is no one that you can approach and consult. It is extremely tough.
HP16	One day, there was this older woman with a walking stick in a government hospital, which was very big and complicated. I saw her holding her insurance card and prescription and she looked confused. Then we assisted her with the process.
HP26	In [hospital name], some voluntary medical students stand in the hospital compound wearing T‐shirts on which the question ‘what can I help you with?’ is printed. They try to identify patients who seem confused and help them with everything.
OP15	We do not understand that for older people, climbing up two or three stair treads is more difficult than climbing up a whole building two times. This is an institutional ignorance I have seen.
HP24	The setup we are in is not a healthcare center setup because it was not built for that.
HP22	When they do the construction part, they don't involve the health professional, and they think of only constructing it. For example, the rooms are not ventilated, and the corridors are very narrow, so you have to make them wider. Disability or older adults were not considered when constructing. They may consult the sub‐city administration people, but not us. I think this is a system problem. People at the top do it to the extent of their knowledge, but the health professional on the ground is the one who will have challenges.
OP18	Most of the hospitals have a shortage of equipment.
HP23	The medical setting does not consider older patients.
HP8	The healthcare setup in our country kills you. You are overburdened with patients, and you do not have enough facilities. The older patients may have something treatable, but, for example, an X‐ray that should take one day might take days.
HP21	Currently, the room that was for chronic and older patients is being used as an emergency ward as well, due to maintenance works. So there might be other patients lying near you when you treat an older patient.
HP24	The beds are placed next to each other with no shutters and no privacy. When we refer older patients to such hospitals, they leave because they want and deserve respect. They have things they want to talk about and things they want to hide. So they lose that respect, and they don't want to be treated.
HP21	Making the hospital environment comfortable for older patients is one thing that should be done.
OP14	From the very beginning, hospitals or clinics should be designed considering the physical conditions of older people. Things must be smooth for older people.
HP16	There must be a few volunteers assigned in such hospitals to help older patients who look confused about where to go in the hospital compounds.

Large and complex healthcare facilities, especially government referral hospitals, were also reported to be difficult to navigate. In such settings, participants described older individuals experiencing confusion and frustration as they moved around hospital compounds without guidance. Some health professionals confirmed that older patients often appeared disoriented or overwhelmed and emphasized the lack of adequate support services to help them navigate these environments. While a few participants noted promising practices in a few facilities, such as the presence of volunteer medical students helping confused patients, these were described as exceptions rather than the norm. Most health professionals agreed that the current hospital infrastructures were not designed with the needs of older adults in mind, and some attributed this to a lack of consultation with health professionals during construction planning. Several respondents, particularly those working in public hospitals, indicated that some facilities were initially designed for other purposes and later repurposed for healthcare delivery.

Some of our participants agreed that the existing medical equipment setup was not convenient for conducting quality care, especially for patients with special care needs in general. This was reflected across both participant groups. When asked about the possible consequences of these challenges, some health professionals were unanimous in the view that the absence of adequate medical facilities negatively affected the care provided to older patients. For example, delays in diagnostics or treatment due to limited resources were seen as particularly detrimental for older patients, who may already be vulnerable due to comorbidities.

A few health professionals argued that the unsuitable infrastructure in health facilities could put the privacy of their older patients at stake. However, this is a general problem of the health care facilities that could affect all patients, although it might disproportionately affect older people since they tend to suffer from more illnesses. In crowded hospital settings, older patients were often examined in shared rooms with little to no privacy, and this was reported by participants as contributing to feelings of disrespect and discomfort among older patients. Health professionals noted that this lack of personal space could discourage older patients from fully engaging with healthcare services or from disclosing sensitive health information. Some participants further gave comments that the healthcare infrastructure should be improved to accommodate patients with limited mobility, including older patients and patients with physical disability. This included not only better architectural designs but also operational changes—such as staffing volunteers or guides to assist patients in navigating large and complex healthcare environments.

### 3.2 Lack of Specialized Care for Older Patients

While infrastructural shortcomings affect all vulnerable patients, older adults often experience these challenges more acutely due to the higher burden of disease and age‐related physical limitations. In addition to infrastructural issues, participants widely discussed the absence of specialized care tailored for older patients (Table 3). All participants stated that there was no specialized care for older patients in Ethiopia. Most of them specifically used the term “geriatric care” and underlined that such specialized care did not exist in health facilities in which they have experience working or getting treatment. Across both participant groups—older adults and health professionals—there was consensus that geriatric‐specific care was lacking in the Ethiopian health system. Many explicitly noted that no geriatric wards or services were available in the facilities they had visited or worked in.

**Table 3:** Illustrative quotes for Theme 2: Lack of specialized care for older patients.

Participants	Quotes
OP9	We do not seem to have geriatric wards in hospitals.
HP15	No special thing is done for older patients.
HP25	As they are older, they want special respect different from others, and actually, they must be treated in a special way.
HP6	Since they are older people, health professionals respect them, and this is also in our culture.
HP19	Older patients are admitted as adults and not classified by age, so their care is not different from others.
HP23	An older patient with diabetes is not the same as an adult or a child with diabetes. The disease has its own headache, and with age, it becomes more problematic because of other age‐related chronic conditions, such as heart problems, hypertension, etc.
HP3	You cannot say ‘go there, do this, do that′ to older patients. Even though the medical discipline says treating all irrespective of age, their age still matters in how you treat them.
HP16	You may face a shortage of materials, such as diapers, for older patients.
OP11	One day, I broke my hip and was taken to [hospital name]. They said I needed hip replacement surgery, but could not find something that fits my size. But since I have UN health insurance, I was lucky and taken abroad.
HP6	For example, the meals hospitals provide for older patients should go with their treatment plan. And they should have space to relax, watch TV, etc. These problems are related to our country′s economic condition.
HP25	The older patients get health services mixed with the young ones. For example, when they are referred, they have to come and go three or four times or be placed in the queue like anyone else. This dissatisfies them and they complain.
HP6	They get treated like everybody else, and that is not comfortable for them when you consider their age, the things they are used to at home, etc.
OP11	I am above 60 years old, but they didn′t give me any special treatment.
OP15	I am getting the same kinds of medicines that I used to be given when I was young. They do not consider my age and medication side effects.
HP21	Treatment services should be adequately available in health facilities.
HP10	In foreign countries, geriatrics treatment is given separately, and it has to be the same here. Their [older patients′] medication, admission, and all should be separate.
OP9	My main recommendation is to have geriatric care services throughout the country. You cannot just leave it up to the health professional to have a heart for older patients. You have to have the care systematized throughout the whole healthcare system.

Several health professionals voiced the need for specialized care services for older patients. All health professionals who commented on this were in agreement that older patients should receive different care simply because of their age, regardless of their health conditions. They argued that care should reflect both a clinical understanding of ageing and the cultural expectation of respect toward older adults. However, participants noted that older patients are often admitted and treated in the same manner as younger adults without differentiation. The unique nature of ageing and the complexity of managing multiple coexisting conditions were raised as a justification for why older patients should not be approached as just another adult patient. Participants further pointed out that clinical approaches often overlook the subtle ways age impacts diagnosis, treatment, and response to care. For instance, a chronic condition such as diabetes was said to manifest differently in an older person due to other concurrent illnesses like hypertension or heart disease. Additionally, it was noted that the communication style and instructions provided to older patients often fail to take into account age‐related factors such as slower mobility, reduced hearing, or lower health literacy.

A few participants from both participant groups also briefly discussed why tailoring care services for the specific care needs of older patients could be difficult. Age‐appropriate medical supplies and equipment, such as adult diapers or prosthetic devices fitted for older individuals, were reported as either unavailable or difficult to access. In extreme cases, patients had to be referred abroad for treatment due to such shortages. Moreover, the lack of supportive services, such as appropriate meals, rest spaces, and recreational options, was raised as an additional challenge and attributed to broader economic limitations faced by the health system.

Several health professionals provided different examples to illustrate how the limited resources and absence of specialized geriatric consultations affected the treatment experiences of older patients. Older patients reported receiving the same medicines or services they had been given when they were younger, with little consideration for how ageing may alter drug metabolism or increase vulnerability to side effects. Health professionals similarly expressed concern that older patients were placed in queues alongside younger patients, asked to navigate complex referral systems on their own, and subjected to processes that failed to account for their age‐related needs. This often led to dissatisfaction and disengagement from the healthcare system.

Within the framework of specialized healthcare services for older patients, several participants also discussed areas of improvement. They asserted that geriatric sections should be established, especially in tertiary hospitals. Some participants referenced international models where older adults receive tailored services from admission through discharge. There was agreement that relying solely on the goodwill of individual health professionals is insufficient. Instead, integrating more geriatric care services into the Ethiopian healthcare system was strongly suggested.

### 3.3 Lack of Competence to Treat Older Patients

Lack of competence to treat older patients was broadly discussed by most of our participants as a major gap in healthcare facilities in Ethiopia (Table 4). The older patient participants' narratives illustrated their experience of getting healthcare services in a context where competence to treat older patients is seriously lacking. A few older patient participants further expressed their assumptions that health professionals might not have received any adequate geriatric training as part of their formal medical education. The responses from the participating health professionals were consistent with the older patients' views in that the health professionals were concerned about the lack of geriatric knowledge and its effect on the healthcare services they provide to older patients. It was also argued that the lack of specialized knowledge could affect the treatments older patients receive. For example, health professionals might not understand that older patients' situations are different and customize their services accordingly. They further elaborated on this by referring to more serious consequences, such as misdiagnosis, and underlined how that could affect older patients' healthcare‐seeking behavior. Although health professional participants remained vague on what it means to understand older patients, their responses are clear in that they underline the lack of expertise in understanding the diseases and healthcare needs of their older patients.

**Table 4:** Illustrative quotes for Theme 3: Lack of competence to treat older patients.

Participants	Quotes
OP10	The health professionals do not look like they are qualified for older patients.
OP1	The health professionals′ training is not clear about how much attention older patients need. They have learnt about mothers, children, diabetics, etc. But the approach to an older person with diabetes and others with the same disease should be different.
HP6	I think there is no geriatric knowledge among health professionals.
HP6	I think that we lack geriatric expertise and treat them as any other patient. I should know how they feel, what to do, etc. They might expect many things because of their age.
HP20	There will be a misdiagnosis and, as a result, they will not come for follow‐ups even if they have to. Why do they waste their time, energy, and money coming now and then if we do not understand them?
OP10	The health professionals need training so that if, for example, a patient with an eye problem comes, they will not just check that and finish. But they understand the whole body and properly communicate what is going on with the family.
OP11	All health professionals, whether they be nurses or highly specialized, especially need ethics training on what to say and how to do things for older patients.
HP1	Even short training on how to treat older patients will be good and can enable us to help these patients better.
HP21	Special training and short courses are necessary to create good relations and communication with older patients, so that our services to them will be better.
HP4	If you have the desire to help older patients, you could just read. If I say I am educated and stop there, neither I nor the patients I help will improve.

Some of our participants further commented on what should be done to address the knowledge and skill gap among health professionals in treating older patients. Older patients suggested that health professionals be educated to take a more holistic approach to care, understanding the whole body and engaging with the patient's family in communication. Some also underlined the need for ethics training to help professionals better interact with older patients with appropriate sensitivity and respect. Similarly, several health professionals also suggested that they need such training. They recommended practical solutions such as short courses and refresher training that would help them build better rapport and communication skills with older patients. Others highlighted the importance of self‐directed learning, emphasizing that continued reading and staying updated are essential to improving the quality of care they provide.

## 4 Discussion

This study explored the gaps in providing healthcare to older patients using qualitative interviews with older adults and health professionals in Ethiopia. To our knowledge, this is the first qualitative study, including older patients and health professionals, that addressed healthcare access challenges among older patients in Ethiopia. The findings are relevant when it comes to patient rights and discrimination, including the issue of leaving no one behind in providing universal health coverage. Universal access to healthcare is an ethical concern [51, 52, 53] and, as the World Health Organization (WHO) states, “a powerful expression of fairness, solidarity and recognition of health as a human right” [54]. Highlighting challenges for the healthcare of older patients such as inconvenient physical environment, low quality and lack of specialized care, as well as lack of geriatric expertise among health practitioners, this study aligns with current literature underlining the lack of universal access to geriatric care in low‐ and middle‐income countries in particular [32, 55, 56] and the low quality of healthcare in general in low income contexts [57, 58, 59, 60]. Our findings offer a deeper understanding of the experiences of older patients and health professionals in receiving and providing care services to a geriatric population, the reasons for the perceived shortcomings, their consequences, and possible solutions. They can be useful for healthcare facility administrators and health professionals working with older patients, particularly in low and middle‐income settings.

Our finding that the infrastructure of healthcare facilities can be challenging for older patients aligns with a growing body of literature coming from several African countries [15, 61, 62, 63] and beyond [64, 65, 66]. The problem is recognized by the WHO [67] and also applies to other patient groups with mobility challenges, irrespective of their age. Our study indicates several reasons for this problem. First, health professionals' opinions in designing facility infrastructures (both inpatient and outpatient facilities for all patients) are insufficiently considered. This also contradicts ethical health leadership [68], which implies that the choice and construction of facility physical structures should be a well‐informed decision by inputs from all relevant stakeholders, particularly healthcare professionals [69, 70]. Thus, it is imperative that the opinions of health professionals—who work at the grassroots—are consulted when designing health programs in general and the physical structures of healthcare facilities in particular.

A second reason is the scarcity of healthcare resources. When resources are limited to make facilities conducive for all population groups, there will be a need to prioritize using different criteria, such as who uses the service the most, etc. As Ethiopia has a predominantly young population (having the second‐largest youth population in Africa), there is a high chance that ageing and disability get down on the priority list. This contradicts the culturally embedded expectations of older patients to be treated with respect in Ethiopia [37]. Arguing why and/or how older adults should go up on the priority list is beyond the scope of this paper; however, our findings could inform any efforts to invent ways to accommodate the healthcare needs of older adults even in resource‐scarce situations.

An additional gap identified in this study, which further illuminates the lack of specialized care for older adults in Ethiopia, is limited geriatric knowledge among health professionals, mainly due to the absence of geriatric training. Other qualitative studies in Ghana [71, 72], South Africa [73], and India [74] reported similar observations and highlighted that limited geriatric training among health professionals impacted the quality of healthcare provided for older patients. On the contrary, and showing the positive relationship between good geriatric expertise and quality of care, a qualitative study conducted among older patients in Sweden showed that participants were confident about geriatric competence among health professionals and satisfied with the care they received [75]. Although the Swedish study cannot be fully comparable with our study, it generally shows that advanced geriatric training could help improve geriatric care.

Even though our study did not broadly discuss how much geriatric content the medical curriculum in Ethiopia has, the responses from a few participants imply the need to provide more geriatrics training to medical students (incorporated into medical teaching) and health professionals (as short professional courses). Low geriatric education in medical fields in several African countries is a big challenge for advancing geriatric care across the continent [76, 77]. Further elaborating on this challenge, a review of older people's healthcare and national policies in sub‐Saharan Africa states that “training and research in gerontology and geriatrics are hardly supported by governments in SSA [sub‐Saharan Africa]” [78]. This leaves older patients behind in public health and gives rise to the ethical concern of justice in healthcare distribution. As this study suggested, incorporating more geriatric care in both medical training and healthcare delivery could be helpful to address these gaps. This is in line with current evidence emphasizing the invaluable benefits of health workforce capacity‐building in transforming healthcare quality [58, 79, 80, 81]. Implementing such solutions could be constrained by knowledge and financial resources. However, some measures could still be taken to integrate old‐age care in academia and health programs. More broadly, promoting more research on the area and engaging international and local actors could help address these barriers.

### 4.1 Strengths and Limitations

This study is among the first qualitative investigations of geriatric healthcare access in Ethiopia and thus, offers a unique contribution to a largely understudied area. Its exploratory design allowed for a broad and in‐depth understanding of the structural, professional, and experiential challenges older adults face. By incorporating the perspectives of both older patients and health professionals, the study provides a rich, multi‐voiced account of the health system's responsiveness to ageing populations. The inclusion of participants from diverse professional backgrounds and lived experiences strengthens the credibility and relevance of the findings. Furthermore, the use of reflexive thematic analysis helped ensure a thoughtful and rigorous interpretation of the data.

While the study offers several important insights, it is not without limitations. For example, due to time and resource limitations resulting from the COVID‐19 pandemic, we did not include interviews with informal care providers (family members, e.g.) of older patients. This might have helped us get more perspectives on some of the concepts. Moreover, due to the qualitative nature of this study, the findings are, as Braun & Clarke assert [46], influenced by our perspectives as those holding and interpreting the data. To ensure analytic rigor, all authors were involved in discussing the codes and analysis process to foster a critical and holistic view of the data and the co‐authors' own assumptions and conceptions. An additional limitation could be the possibility of courtesy bias among participants. However, we believe this limitation was kept minimal since the interviewer (KMM) is from the study country (hence limited cultural barriers) and took time to build rapport mainly through ice‐breaking. Moreover, the sampling strategy is that it only includes older adults who have already used healthcare services. This leaves out those with unmet medical needs, meaning barriers such as challenges at home or getting to a facility are overlooked. Including their experiences could have provided a more complete and nuanced understanding of systemic access challenges. A final limitation could be that the data, since it is from an urban setting, may not adequately reflect geriatric care challenges in rural settings. However, in an urban context, we would expect better care accessibility than in rural areas. Thus, the reported problems have to be taken seriously, as we would anticipate even more shortcomings to be reported from rural settings.

## 5 Conclusion

This study presented a qualitative analysis of current gaps in universal access to healthcare among older patients in Ethiopia. Most of the gaps identified in this study may also affect other patient groups, e.g., patients with physical or mental disabilities, irrespective of age. However, older patients represent a very large and growing patient group, making these gaps increasingly urgent from a growing number of patients. The findings inform preparedness and health policy efforts tailored to older adults in Ethiopia. As the older population in the country continues to grow fast, there should be more efforts from the Ethiopian government and other stakeholders to, among other things, include geriatric departments in health facilities and advance geriatrics training in existing medical teachings. Further studies could review the current medical curricula to investigate the availability of geriatric content and ways of its integration. Many of the findings could be similar to those in other contexts as well, and thus, further studies could also build on our findings and investigate more challenges peculiar to the healthcare context of Ethiopia. For example, this study briefly addresses cultural aspects of caring (such as respect) and the role of age in relationships of care. This could be investigated further, together with other cultural aspects of caring within the Ethiopian healthcare context.

## Author Contributions

Kirubel Manyazewal Mussie conceived the project under the supervision of Mirgissa Kaba, Bernice Simone Elger and Jenny Setchell. Kirubel Manyazewal Mussie conducted the interviews with support from Mirgissa Kaba in facilitating the data collection. Bernice Simone Elger, Jenny Setchell, Mirgissa Kaba and Kirubel Manyazewal Mussie were involved in commenting on and revising the interview guides. Bernice Simone Elger, Mirgissa Kaba and Kirubel Manyazewal Mussie were involved in checking the quality of the transcripts. Kirubel Manyazewal Mussie analysed the data with inputs from Jenny Setchell and Bettina Zimmermann and prepared the first draft. All authors revised the drafts and approved the final manuscript.

## Ethics Statement

This study received ethical approval from the Ethics Committee of the University of Basel (Switzerland) and the City Government of Addis Ababa Health Bureau (Ethiopia).

## Conflicts of Interest

The authors declare no conflicts of interest.

## 1 Transparency Statement

Kirubel Manyazewal Mussie affirms that this manuscript is an honest, accurate, and transparent account of the study being reported; that no important aspects of the study have been omitted; and that any discrepancies from the study as planned (and, if relevant, registered) have been explained.

## Supporting information

Additional File 1. Summary of interview guides and probes.

## Data Availability

The data used for this study cannot be publicly available due to the risk of compromising the individual privacy of study participants. However, the transcripts relevant to this paper can be obtained from the corresponding author upon reasonable request. Kirubel Manyazewal Mussie had full access to all of the data in this study and takes complete responsibility for the integrity of the data and the accuracy of the data analysis.
