# Supplementary material for: “We Do Not Seem to Have Geriatric Wards”: A Qualitative Analysis of Gaps in Healthcare Access Among Older Patients in Addis Ababa, Ethiopia
Source: Health Sci Rep. 2025 Nov 5;8(11):e71449. doi: 10.1002/hsr2.71449 (PMC12588954; doi:10.1002/hsr2.71449)
Supplement: Supplementary file 1 — Additional File 1. Summary of interview guides and probes. [file HSR2-8-e71449-s001.docx]

**Summary of the interview guides and probes**

**Interview guide for older adults**

1. Introduction and demographic information

- Please tell me about yourself, including your age, work or activities you do, family condition, etc?

1. Getting medical care in hospital settings

- How would you describe your health condition in general?
- Can you tell me about your last visit to a health care facility? How was it?
- How comfortable are you visiting health care facilities?
- How do you describe the accessibility and quality of the health care services for you or other older patients?
- How comfortable are the hospital buildings for you?
- How do you describe the knowledge and competence of health professionals to treat older patients?

**Interview guide for health professionals**

1. Introduction and demographic information

- Please tell me about yourself, including your age, qualification, work experience, etc.

1. Experience in providing medical care for older patients

- What are the differences in general, if any, between treating a younger patient and an older patient?
- How do you describe your or health professionals competence to treat older patients?
- How do you describe the health conditions of older patients in general?
- What special resources or skills are needed to treat an older patient?

1. Describing the suitability of health care facilities for geriatric care

- How do you describe the physical setting of the facility you currently work in or know of?
  - How is that for older patients?
- How does the health facility physical setup affect the quality of health care services you provide for older patients?
